# Emotional, Cognitive and Self-Enhancement Processes in Aggressive Behavior After Interpersonal Rejection and Exclusion

**DOI:** 10.5964/ejop.v11i4.934

**Published:** 2015-11-27

**Authors:** Joanna Rajchert

**Affiliations:** a Institute of Applied Psychology, The Maria Grzegorzewska University, Warsaw, Poland; Aalborg University, Aalborg, Denmark

**Keywords:** exclusion, rejection, readiness for aggression, aggressive behavior

## Abstract

The relationship between exclusion or rejection and aggression is already well documented, but there is still a debate about the mechanisms that underlie this effect. In two studies we focused on the propensity to react aggressively (readiness for aggression) on the bases of emotional, cognitive or self-enhancement (personality-immanent) processes. In both studies we first measured readiness for aggression and then ego-depleted participants. Next, in Study 1 we excluded participants (n = 96) using an online ball throwing game and measured displaced aggressive behavior - intensity and duration of an unpleasant noise administrated to a stranger. In Study 2 participants (n = 140) were rejected by a peer on the basis of an interview that they gave and then could retaliate by reducing peer's chance for getting a job. The results show that exclusion effect on displaced aggression was moderated by cognitive readiness for aggression, while rejection effect on retaliatory aggression was shaped by emotional and personality-immanent readiness for aggression as well as ego-depletion. The results were discussed in light of the strength model of self-control by Baumeister, Vohs, and Tice (2007).

A number of studies (e.g. Baumeister, Brewer, Tice, & Twenge 2007; DeWall, Twenge, Bushman, Im, & Williams, 2010; Twenge, Baumeister, Tice, & Stucke, 2001) show strong relationship between interpersonal rejection or exclusion with aggressive behavior. Different mechanism were proposed as a base of this relationship (for review see: Leary, Twenge, & Quinlivan, 2006). Most researchers agree that these mechanisms are connected with various aspects of control: self-control defined as a capacity or strength (Muraven & Baumeister, 2000) or feeling of control over situation (Warburton, Williams, & Cairns, 2006). Rejection diminishes motivation for self-control because the connection with others are broken. Rejection also force people to attempt to manage difficult and unpleasant emotions, what exploits the self-control resources leading to increase of impulsive and automatic reactions. Exclusion is also related to feeling of loss of control over situation, thus aggressive behavior might be a way to regain a control. Research shows also that experimental reducing (by inhibiting impulses) or boosting a capacity for self-control (by sugar consumption) and also restoring the feeling of control over environment (by controlling the noise level) influence aggression in expected way (Gailliot et al., 2007; Warburton, Williams, & Cairns, 2006).

In two studies we intended to explore whether three different mechanism at the base of aggressive behavior interact with the rejection or exclusion and shape the aggressive manifestation. As Fraczek suggested (e.g. Frączek, Konopka, & Dominiak-Kochanek, 2015) people differ as to the propensity to base their aggressive behavior on particular mechanisms: emotional, cognitive or related to the self-representing one's needs and motivations. He proposed that Emotional - Impulsive readiness (E-IR) for aggression is a quite stable tendency to react with aggression on the basis of emotional outburst. This propensity is related to low trait self-control, emotionality and emotional reactivity and higher anger. This type of readiness is related to temperamental propensities, mostly emotional reactivity (Frączek, Konopka, & Smulczyk, 2013). Aggressive behavior based on E-IR is limited in time and frequently violent, as it is a manifestation of angry reaction to aversive stimuli and provocation. Women demonstrate higher E-IR than man in Poland and Uruguay, but no sex difference is observed in China and Spain and in the United States women were higher on E-IR than men (Frączek et al., 2013; Rajchert & Frączek, 2012). It can be then assumed that apart of different social roles performed by men and women that shape aggressive manifestations (Eagly & Steffen, 1984) also cultural factors that influence the process of socialization of men and women are important predictor of this type of readiness.

Second pattern of readiness, Cognitive - Habitual Readiness (C-HR) on the other hand is a proclivity for aggression based on well-developed net of aggressive schemes represented by behavior patterns related to social role or profession. Cognitive processes, such as: social perceptions, anticipation and attribution, have a crucial role in H-CR. This class of readiness is responsible for habitual and planned aggressive behavior, which is perceived as approved and legitimized, important, obvious and necessary part of social relations. Habitual - Cognitive Readiness is weakly related to temperamental features and sex differences in this pattern of readiness are stable across countries with men scoring higher than women.

There is also a third pattern of readiness, personality-immanent readiness (P-IR) for aggression possible, when one feels pleasure and satisfaction as a consequence of aggressive act. People who can be characterized by this type of readiness use aggressive behavior as a way of enhancing their self-esteem and control over the situation. Studies show that P-IR is related to Eysenk's Psychopathy scale and is more prevalent among men in different cultural settings (Frączek et al., 2013; Rajchert & Frączek, 2012).

Baumeister and colleagues propose in strength model of self-control (e.g. Baumeister, Vohs, & Tice, 2007; Muraven & Baumeister, 2000) that self-control is a control over the self by the self and that it can be depleted or consumed by previous acts of effortful control. Repeated exertions of self-control, for example regulation of negative emotions experiences after ostracism, deteriorate the self-control capacity, that is a limited resource, leading to worse performance on other self-control tasks. Furthermore Baumeister and colleagues argue that people differ in their base self-control strength or resources. We think that people with predisposition for aggression on the basis of emotional, cognitive or self-imbibed mechanisms also differ with regard to their self-control strength or the capacity to inhibit the automatic reaction in different situations. Individuals with high E-IR are the least able to self-regulate their emotions because of their low self-control strength, so direct, painful rejection episode should anger them and deplete their capacity to inhibit aggressive retaliatory response toward someone, who rejected them. However, if the exclusion situation would not be emotionally disrupting enough to cause angry outburst, than E-IR should not influence aggression level. Quite different effects could be predicted for high C-HR in those two situations. People with cognitive propensity for aggression hold many beliefs approving aggressive behavior. Frączek's (2015) conceptualization posits that they see aggression as normal and frequent in their environment and develop a hostility bias in evaluation of social situation. Research shows that this hostility bias may underlie aggressive behavior after exclusion because people with many aggressive scripts tend to see ambiguous behavior as aggressive and react accordingly to their interpretation (DeWall, Twenge, Gitter, & Baumeister, 2009). Then we could expect that the hostility bias would be the most noticeable, when exclusion will be less severe and obvious (more ambiguous), for example when rejection is not communicated directly but only deduced from the observation of other people's ostracizing behavior. Ambiguous acts leave much more space for subjective interpretation that may engage hostile cognitions (characteristic for high H-CR individuals) leading to more aggression (e.g. Crick & Dodge, 1996; Dodge & Coie, 1987). With regard to high P-IR individuals, we think that they are prone to aggression without any instigation, so being rejected or excluded should not matter for their aggression level. Rather they might be more aggressive toward anybody and anytime, when for example they need to boost their self-esteem or they feel bored or unhappy. Although high P-IR individuals are thus not particularly susceptible to situational triggers of aggression, and can act aggressively even without provocation, they would also respond with an increased aggression if a provocation derogates their self-esteem and they perceive aggression as a mean for enhancing their self-appraisal.

To strengthen the differences between people with three readiness types we also depleted their self-control strength. Experimental ego-depletion cause people to behave more automatically and cause more aggression after provocation (Muraven & Baumeister, 2000; Schmeichel & Baumeister, 2004), so we hoped that the interaction effect of readiness for aggression and exclusion on aggression will be stronger when individuals would be egodepleted.

To test our assumptions we conducted two studies in which we first measured readiness for aggression, next ego-depleted participants, then in Study 1 excluded or included them and measured their displaced aggressive reaction and in Study 2 rejected or accepted them and gave them a possibility to be aggressive toward someone who rejected them.

## Study 1

In Study 1 we sought to verify first, whether exclusion would cause more displaced aggression than inclusion and second whether this effect would be shaped by level of readiness for aggression. It was hypothesized that less direct and severe exclusion in contrast to rejection, would be interpreted as more antagonistic behavior by high C-HR individuals but not by less C-HR participants and the effect would be more pronounced after ego-depletion. We also hypothesized that P-IR would be positively related to aggression level among excluded and included individuals as well. In this study we also controlled for the level of E-IR, but did not hypothesize it to be a significant moderator of exclusion - aggression relationship or have a main effect on aggression either since the exclusion was indirect.

### Method

#### Participants and Procedure

Ninety six Polish students aged from 18 to 25 years old (*M* = 21.35, *SD* = 1.71; 48 men and 48 women) from different universities and schools participated in the study voluntarily, without any monetary incentive.

First participants completed the Readiness for Interpersonal Aggression Inventory (RIAI, Frączek, Konopka, & Smulczyk, 2009) measuring three types of readiness for aggression. The inventory consists of 30 statements with yes and no answering possibility. Three scales measure E-IR, H-CR and P-IR. Each scale includes 10 items. Questionnaire has a good predictive validity and internal consistency ranging from α = .76 for C-HR to α = .67 for E-IR. In the current study consistency was even better: for E-IR alpha = .75, for H-CR alpha = .84 and for P-IR alpha = .75. Next participants were ego-depleted by reading the words representing basic colors printed with incompatible ink color. Such a tusk requires participant to inhibit competitive reaction to name the color of the ink instead of reading the word list. In the control condition participants were reading the words naming colors printed in black ink on white paper. Both groups were reading the lists in the presence of the experimenter for 5 minutes. When finished, participants completed for the first time the Positive and Negative Affect Schedule by Watson, Clark, & Tellegen (1988) in polish adaptation by Brzozowski (2010) to check whether the manipulation that followed was effective. To manipulate the feeling of exclusion individuals were playing the Cyberball game on a computer (Williams & Jarvis, 2006). In this game participants throw a virtual ball to two other participants. In fact the game is preprogrammed and no other players exist. In the exclusion condition participants get only 2 first throws and for about 3 minutes observe how two other people are playing with each other. In the inclusion condition participants obtain as many throws as others. After the Cyberball participants completed PANAS again and aggressive behavior was measured by Competitive Reaction Time game (CRT; DeWall et al., 2010). In this computer program, individuals compete with other participant (in Study 1 it was someone, who was not previously playing Cyberball with participant) in fast clicking on the rectangle. The person who was faster in each trial punishes the opponent with a noise by setting the duration and intensity from 0 to 10. The participant always wins the first trial and has to set the noise level for the opponent, which is used as an index of unprovoked aggression. After CRT participants were asked whether they believed in the experimental mask, next thanked and debriefed.

### Results

The exclusion manipulation was effective. Participants declared that they felt less included after exclusion than after inclusion (*M* = 2.47, *SD* = 1.98 vs. *M* = 5.89, *SD* = 2.04, *t*(95) = 8.22, *p* < .001). Excluded participants also noticed that only a small percentage of all throws was directed to them (9%, *SD* = 8.14 vs. 38%, *SD* = 12.20, *t*(95) = 13.60, *p* < .001). Excluded and included group also differed in positive and negative emotions. The analyses were conducted using a repeated measures ANOVA separately for positive and negative affect measured by PANAS. The interaction term between time of measurement (directly before and after the manipulation procedure) was significant for positive affect, *F*(1,95) = 26.50, *p* < .001, partial *η*^2^ = .22. Post hoc analysis showed that following manipulation, excluded individuals declared less positive affect, *M* = 25.38, *SD* = 1.18, than included, *M* = 33.10, *SD* = 1.35, *t*(47) = 3.99, *p* < .001, and participants from the excluded group had higher positive affect before manipulation, *M* = 32.38, *SD* = 1.18, than after manipulation, *t*(47) = 5.93, *p* < .001. No other post hoc comparisons yielded significant differences. The interaction also was significant for negative affect, *F*(1,95) = 6.38, *p* < .05, partial *η*^2^ = .06. After manipulation, excluded participants had more negative affect, *M* = 14.35, *SD* = 5.96, than included, *t*(47) = -2.38, *p* < .05, and included participants had more negative affect before manipulation, *M* = 13.17, *SD* = 3.96, than after, *M* = 12.02, *SD* = 3.16, t(47) = 2.19, *p* < .05. There were no other significant differences for negative affect. Most participants did not believe that they had played the Cyberball game with real people (we used a 7-point Likert scale, where 7 represented very strong belief), *M* = 2.36, *SD* = 1.64. What is more, many participants were not convinced, that they have played with real people in CRT (*M* = 2.48, *SD* = 1.73 on a 7-point scale).

To test whether manipulation of exclusion, ego-depletion and gender of participants affected aggression, 2 x 2 x 2 ANOVA was conducted. Results showed no significant differences in displaced aggression due to manipulation or gender.

Next analysis of correlation between aggression and readiness for aggression was implemented (Table 1).

**Table 1:** Pearson r Correlation Indices Between Readiness for Aggression and Displaced Aggression

Measures	1	2	3	4
1. Displaced Aggression	-	.22*	.26*	.33**
2. E-IR		-	.25*	.27*
3. C-HR			-	.69**
4. P-IR				-

*p < .05. **p < .001.

The readiness for aggression patterns were inter-correlated in a predicted direction, as in other studies (Frączek et al., 2013). When other types of readiness were not controlled, all patterns of readiness were positively related to aggression.

To verify the interaction hypothesis a series of hierarchical multiple regression analyses for each readiness for aggression pattern were conducted. The final model, presented in Table 2, included all main and interactive effects and predicted 24% of variance in displaced aggression (*p* = .032).

**Table 2:** Hierarchical Multiple Regressions Models for Readiness for Aggression on Displaced Aggressive Behavior

Variables	Final Model		
	*B*	*SE*	*p*
Constant	8.37	1.15	.000
Exclusion (EX), Inclusion = 0	1.13	1.41	.425
Ego-depletion (ED), No ED = 0	1.07	1.36	.443
Gender, Women = 0	0.17	1.18	.886
E-IR	-0.02	0.82	.982
C-HR	-0.86	0.93	.357
P-IR	1.86	1.03	.075
EX x ED	-1.46	2.11	.490
E-IR x EX	0.43	1.35	.747
E-IR x ED x EX	1.25	1.46	.395
C-HR x EX	4.01	1.73	.023
C-HR x ED x EX	-4.52	2.57	.082
P-IR x EX	-2.07	1.68	.221
P-IR x ED x EX	2.09	2.22	.348

**Figure 1:**
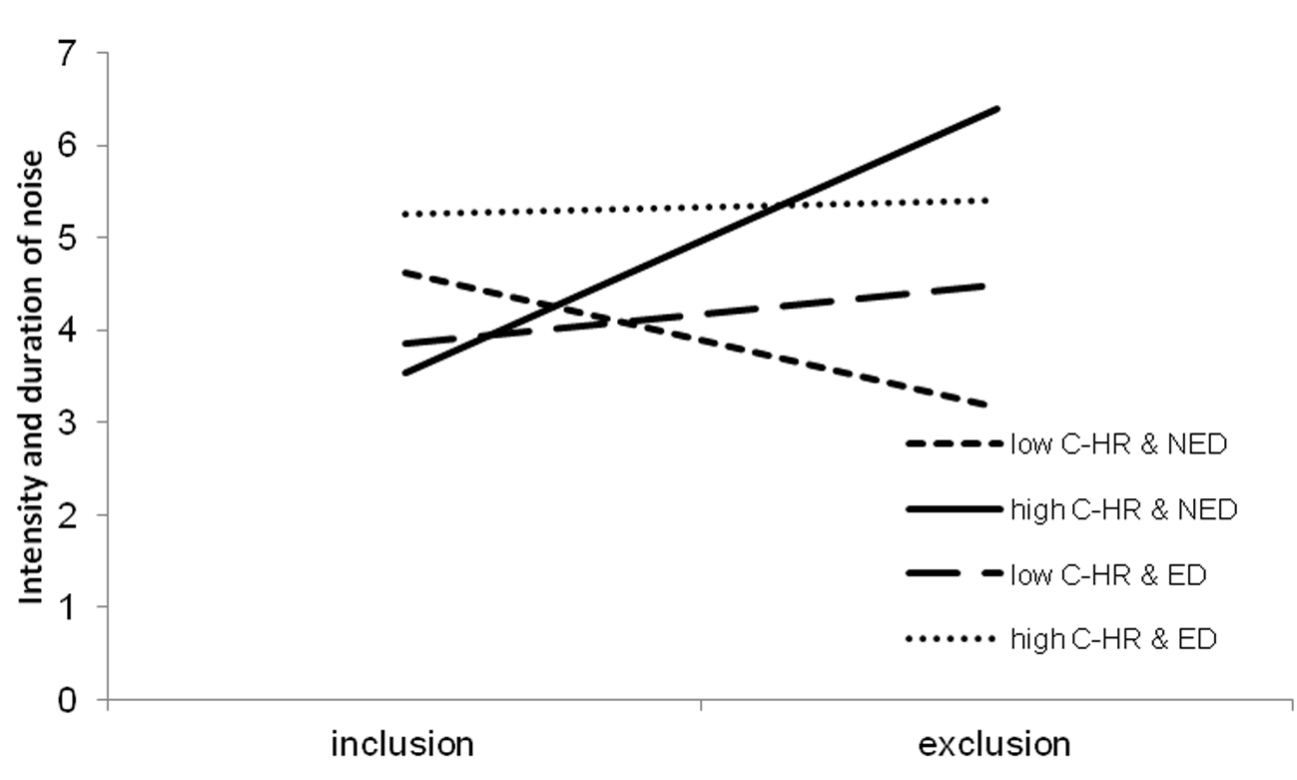
Interaction effect of exclusion, ego-depletion (ED; NED, no ego-depletion) and C-HR on displaced aggression. Slopes are plotted at the ±1 SD.

Results were as we expected. Controlling for all other variables in the model, P-IR was positively related to aggressive behavior at the level, that was close to the significance (*p* = .075). Also interaction between C-HR and exclusion was significant and 3-way interaction of C-HR, Ego-depletion and exclusion was close to significance. Further analysis allowed for interpretation of the interaction effects. We tested the moderated moderation model (Hayes, 2013) in which aggression was predicted by exclusion and this relationship was moderated by C-HR. Ego-depletion moderated the interactive effect of exclusion and C-HR on aggression. It turned out that the interaction term was significant only when participants were not depleted (*b* = 2.15, *p* = .002). Moreover only participants who were high on C-HR and were not depleted differed in aggressive behavior between exclusion conditions (*b* = 2.84, *p* = .003). Means were presented in Figure 1.

### Discussion of Study 1

We assumed, based on other research results in which identical procedures for manipulation of exclusion (e.g. Warburton, Williams, & Cairns, 2006) and measurement of aggression (DeWall, Twenge, Bushman, Im, & Williams, 2010) were used, that exclusion in the Cyberball game would cause more displaced aggressive behavior than inclusion, but results of Study 1 contradicts this prediction. Apparently the feeling of exclusion that was experienced by participants in our study was not strong enough to cause a main effect of exclusion on aggression. For example DeWall et al. (2010) observed increase in aggression measured by CRT among ostracized in Cyberball participants. Also at least two meta-analysis indicated that Cyberball procedure cause less positive and more negative emotions, diminishes basic need satisfaction and increase aggressive reaction toward a guilty and also an innocent other (Gerber & Wheeler, 2009; Hartgerink, vanBeest, Wicherts, & Williams, 2015). It was also indicated that ostracism induced by Cyberball procedure may lead to more positive social behavior or at least behavior that is motivated by the need to reconcile and reconnect with others (Gerber & Wheeler, 2009). Thus, the behavior after exclusion is not always directed against others and motivated by the desire to hurt a target. When other conditions favor such behavior (for example there is a possibility of further contact with the ostracizing partner or the relationship is valued), ostracism may be followed also by pro-social behavior (Smart Richman & Leary, 2009). However, we are not aware of any study that used either Cyberball or CRT game procedures in Polish sample. Polish students in Study 1 were very suspicious with regard to the procedure of exclusion manipulation and aggression measurement, which might have influenced the significance level of the effect. On the other hand a study by Zadro, Williams, and Richardson (2004) showed that mean indices of four basic needs fulfillment (feeling included, feeling of control, meaningful existence and positive self-esteem) did not differ between people who received explicit information that they would play with a computer and participants who were told that they would play with other people. In both groups individuals declared lower needs satisfaction in exclusion than in inclusion condition. In our study participants also declared that they felt less included and had less positive mood after exclusion than after inclusion. We do not know however if the procedure would still be strong enough to produce aggression, when people knew that they were excluded by a computer. In any case participants were also suspicious about CRT and that could finally turn the tide to less aggressive behavior. In future studies it would be necessary to use other ostracism procedures, that would not induce suspiciousness among participants.

Although we could not show that exclusion by Cyberball intensified aggressive behavior among all participants, we demonstrated that at least part of them reacted more aggressively than others. Results indicated, along with our prediction, that the effect of exclusion on aggression was stronger for high C-HR than for low C-HR participants. Contrary to our expectation, ego-depletion did not boost this effect but rather reduced the difference in aggression between excluded and included individuals with high C-HR by increasing aggression among included participants. Ego-depletion causes more automatic and less controlled behavior, thus it could activate cognitive hostility bias among high C-HR participants even after inclusion, what leaded them to more aggression.

The Study 1 also showed, as predicted, that P-IR was a predictor of aggression. This relationship was positive and moderate in simple correlation analysis but weak, when all other patterns of readiness for aggression and their interaction with experimental conditions were taken into account. Still, we can conclude that people, who often experience satisfaction and pleasure as a consequence of being aggressive act that way even when they are included and might be in general more aggressive than people, who do not feel pleasure in harming others.

## Study 2

In Study 2 we wanted to verify hypothesis that (1) experience of rejection would cause more retaliatory aggressive behavior toward the rejecting partner and that (2) this effect would be moderated by E-IR. I was also predicted that (3) ego-depletion would strengthen the interaction effect of E-IR and rejection on aggression. We based our hypothesis on the assumption that sever and unfair rejection - straight forward information about exclusion from a relationship based on insufficient or scarce knowledge about a rejected person, would more greatly affect those, who have problems with emotion regulation and control of impulses. Rejection would be in this case a provocation that would evoke aggression toward the rejecting person among all participants, but the most aggressive would be those with the least control capacity (high E-IR individuals), especially after ego-depletion which would diminish their ability to self-control even more. As in Study 1, we also predicted that P-IR would be a positive predictor of aggression irrespectively of experimental conditions.

### Method

#### Participants and Procedure

Study was conducted in two parts. During the first part 140 Polish students of pedagogy (17 men and 123 women) aged from 18 to 25 (*M* = 21.35, *SD* = 1.71) completed Readiness for Interpersonal Aggression Inventory in groups during the introductory psychology course class. The internal consistency of RIAI scales in Study 2 were good with α = .75 for E-IR, α = .70 for C-HR and α = .60 for P-IR. One week later students were invited to the laboratory for the second part of the study which was conducted individually, that was presented to them as a separate experiment on interpersonal relations via computer and media. This part of the procedure began with the mood measurement using PANAS. Next participants were told that they would exchange video recordings with a peer to decide whether they want to work together later. First, all participants watched the same video of a female answering 3 questions (e.g. "How do you find the food in your school's cafeteria?"). Participants were to decide whether they want to work with the person from the video later. After they made their decision, they were recorded answering the same 3 questions. Their decision and recording was then delivered by the experimenter to the female peer, who never existed. During the time when experimenter was delivering the message to the peer, half of the participants were ego-depleted. Ego-depletion procedure was based on procedure used in Stucke and Baumeister (2006) study. Participants received a text from National Geographic divided in 2 parts and printed on both sides of a paper sheet. At the first page was the instruction to circle all letter a in the whole text which counted 144 words. We wanted to provoke an automatic response to circle the letter a. On the next page participants were also to find and circle letter a, but only in those words which contain also letter m, b or k. This part of the text counted 131 words and participants had to inhibit previously learned automatic response to letter a. The second half of participants that was not ego-depleted circled all the letter a in both parts of the text, so they did not have to break their automatic response. When participants finished that task, the experimenter delivered the feedback from the peer. In acceptance condition participants could read that "The interview was cool! I would really like to work with you later.". In rejection condition the information was: "This interview was poor! I would never want to work with you.". After receiving the feedback participants completed PANAS again and were told that the experiment have finished. Before they left, experimenter told that the peer with whom they just have interacted wanted to work as an experimenter's assistant and needed to collect as many ratings as she could. All participants agreed to complete 10-item survey (e.g., ‘You can rely on this person’; ‘This person is friendly’) on a scale ranging from 1 = completely disagree, to 10 = completely agree, rating the peer's suitability for the job. Next participants were asked question measuring suspicion, thanked and debriefed.

### Results

First we conducted analysis of difference in negative and positive affect to check for the effectiveness of the manipulation. As in Study 1 the analyses of repeated measures ANOVA were run on positive and negative affect scores. The interaction between rejection manipulation and time of measurement was significant for positive, *F*(1,139) = 17.93, *p* < .001, partial *η*^2^ = .12, as well as negative affect, *F*(1,139) = 12.23, *p* < .01, partial *η*^2^ = .08. Further analysis revealed that among included participants positive, *t*(47) = 5.93, *p* < .001, as well negative affect, *t*(47) = 5.93, *p* < .001, differed between measurements with lower positive, *M* = 27.58, *SD* = 7.06 vs. *M* = 31.11, *SD* = 7.43, and higher negative affect, *M* = 16.30, *SD* = 4.72 vs. *M* = 12.76, *SD* = 3.50, before than after manipulation. Following manipulation, excluded participants also declared more negative affect, *M* = 15.97, *SD* = 6.73, than included, *t*(47) = 5.93, *p* < .001. Post hoc tests showed no other significant differences. Participants were also asked whether they believed that they have interacted with a real person. They were giving an answer on a 7-point scale, where 7 signified that they completely believed in the experimenter's story'. Results showed that participants were not very suspicious and by average believed that they interacted with a real person (*M* = 4.27, *SD* = 2.01) and completed the job evaluation survey for the real person (*M* = 4.13, *SD* = 2.04).

Next correlation analyses were conducted for aggression and readiness for aggression, which showed that only H-CR was related to retaliatory aggressive behavior. In this analysis also P-IR was related to C-HR. In this analysis only P-IR was weekly related to C-HR. Pearson r indices are presented in Table 3.

**Table 3:** Pearson r Correlation Indices Between Readiness for Aggression and Retaliatory Aggression

Measures	1	2	3	4
1. Retaliatory Aggression	-	.10	.17*	.01
2. E-IR		-	.05	.00
3. C-HR			-	.34**
4. P-IR				-

*p < .05. **p < .001.

In the next step we verified the interaction hypotheses using a series of multiple hierarchical regression analyses. The final model is presented in Table 4.

**Table 4:** Hierarchical Multiple Regressions Models for Readiness for Aggression on Retaliatory Aggressive Behavior (the More Positive Evaluation was Granted the Less Aggression)

Variables	Final model		
	*B*	*SE*	*p*
Constant	7.40	.30	.000
Rejection (R), Inclusion = 0	-2.27	.42	.000
Ego-depletion (ED), No ED = 0	0.23	.45	.599
Gender, Women = 0	0.45	.51	.380
E-IR	0.09	.21	.672
C-HR	0.08	.25	.975
P-IR	0.02	.24	.995
R x ED	-0.00	.61	.995
E-IR x R	0.02	.36	.951
E-IR x R x ED	-1.11	.49	.027
C-HR x R	-0.54	.47	.251
C-HR x R x ED	0.18	.47	.703
P-IR x R	1.01	.51	.049
P-IR x R x ED	-0.99	.54	.072

The final model explained 39% of variance in retaliatory aggression (*p* < .001). The strongest and highly significant effect was obtained for rejection manipulation (*b* = -2.27). Participants were more aggressive (gave the peer less positive recommendation for the job) in the rejected group than in the accepted group.

**Figure 2:**
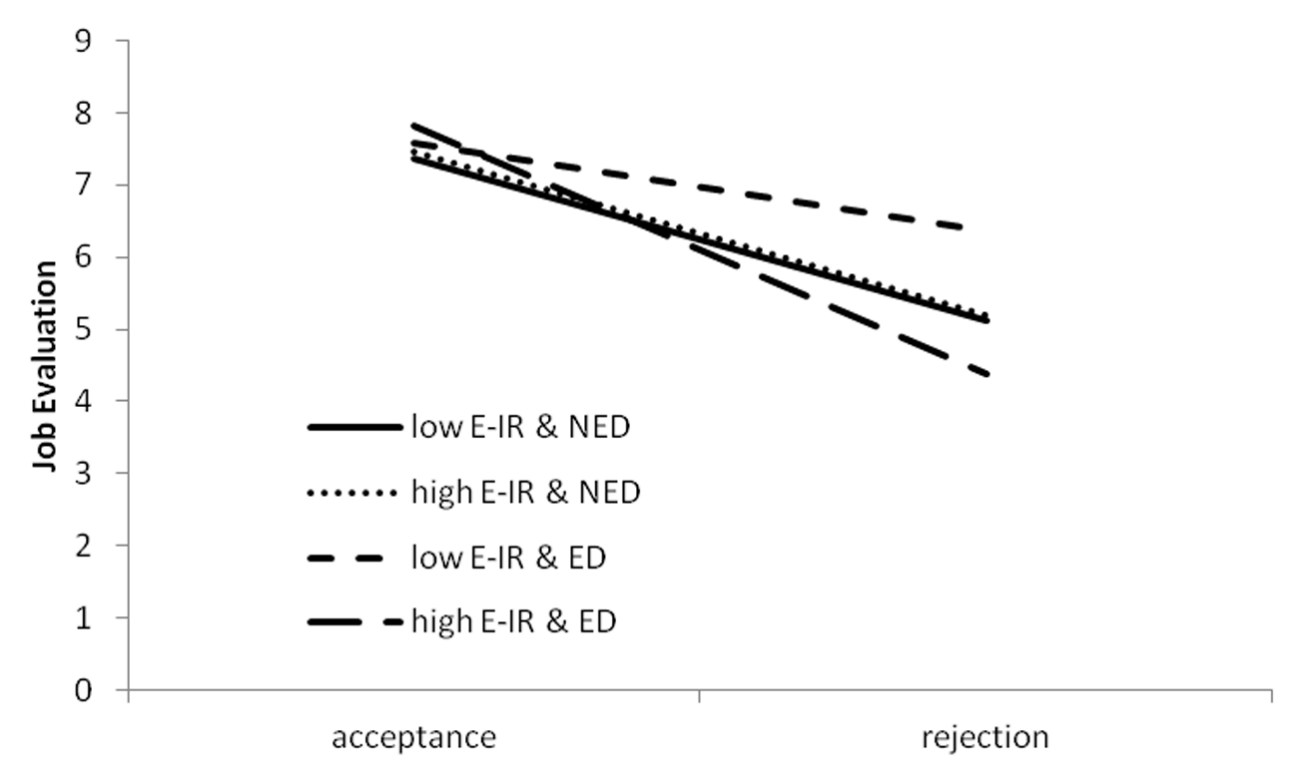
Interaction effect of rejection, Ego-Depletion (ED; NED, No Ego-Depletion) and E-IR on aggression. Slopes are plotted at the ±1 SD.

Furthermore there were two significant interaction effects and one interaction effect was reaching significance level. We hypothesized to find an interactive effect of E-IR and rejection on aggressive behavior, but we supposed that this effect would be more pronounced among people, who were also ego-depleted. Thus, we hoped that both interaction effects for E-IR, two-way as well and three-way, would be significant. It turned out that only a 3-way interaction effect was significant. Interpretation of this effect showed that the effect of rejection on aggression differed for low and high E-IR, but only when participants were ego-depleted (*b* = -1.11, *p* = .026), but not when their self-control strength was left as it was. We also observed that the effect of rejection was highly significant in all groups beside one - participants with low E-IR, who were also ego-depleted (*b* = -1.21, *p* = .066). Mean aggression scores were presented in Figure 2.

**Figure 3:**
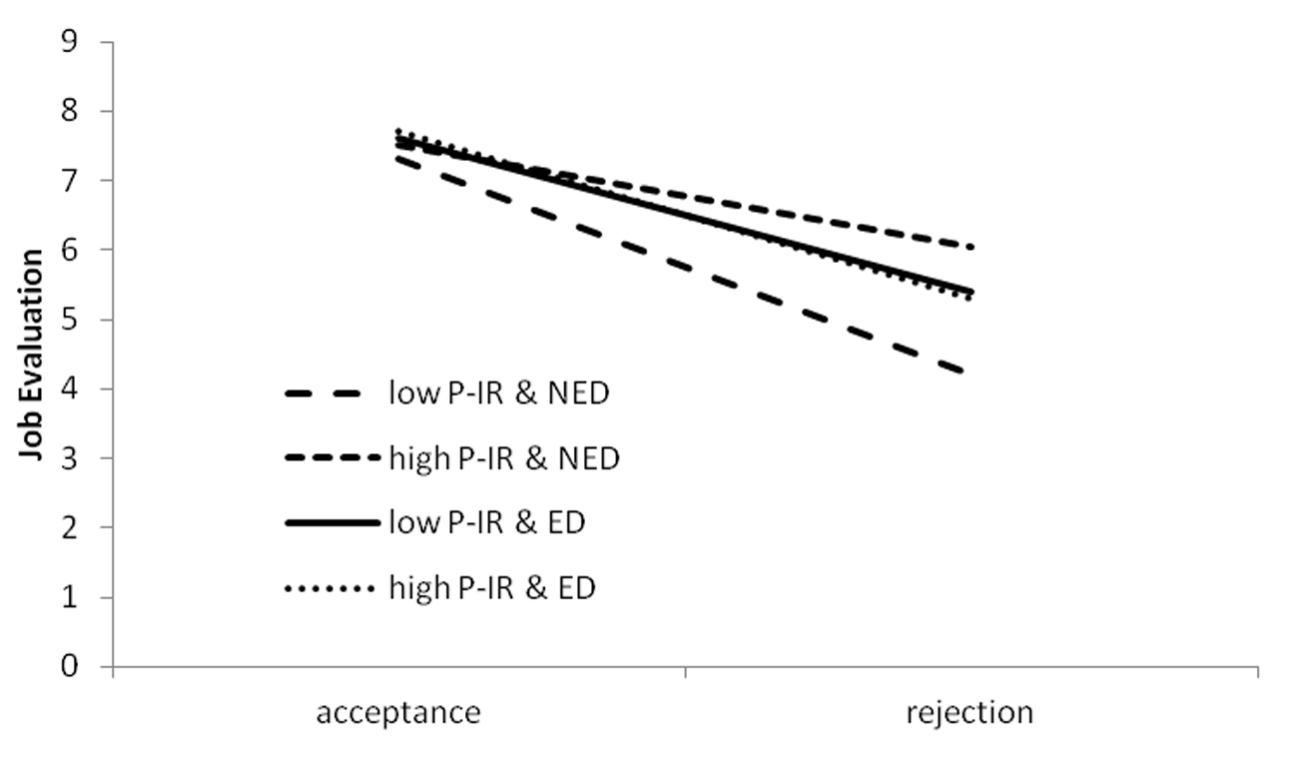
Interaction effect of rejection, Ego-Depletion (ED; NED, No Ego-Depletion) and P-IR on aggression. Slopes are plotted at the ±1 SD.

In addition interaction effect of P-IR and rejection was significant and the three-way interaction was close to significance. Interpretation of these effects indicated that P-IR and rejection interaction was more significant when there was no ego-depletion (*b* = 0.94, *p* = .098) than when there was ego-depletion manipulation implemented (*b* = 0.11, *p* = .805). Effects in all groups were very strong ranging from -3.20 to -2.20, *p* < .001. The least significant (although still quite strong) effect was observed for high P-IR participants when they were not ego-depleted (*b* = -1.46, *p* = .033). Means were presented in Figure 3.

### Discussion of Study 2

Results confirmed the prediction that rejection would cause more retaliatory aggression than acceptance. This result was not surprising, if we consider the procedure of rejection that was giving the impression that the interaction partner was rude and outwardly aggressive on the basis of a very scarce information about the participant. Such provocative rejection must have angered and irritated almost everyone leading to retaliation, what was showed in Study 2. Research using similar procedures for inducing feeling of rejection as well as for measurement of aggression gave comparable results (e.g. Stucke & Baumeister, 2006). Although the interactive effect of E-IR and rejection was not significant - low and high E-IR participants were not different in their reaction to rejection and acceptance - ego-depletion made the difference visible. When ego-depleted, participants with low E-IR were able to restrain their aggressive response after rejection, while those with high E-IR became even more aggressive. We think that ego-depletion reduced self-control that was diminished already by rejection but only among individuals with high E-IR, what resulted in outburst of aggression. Contrary to expectation, participants with low tendencies for emotionally driven aggression controlled themselves more after ego-depletion than without ego-depletion. It is possible that depletion strengthen the dominant, automatic reaction that would be the inhibition of aggressive impulses among low E-IR participants. This inhibition would consequently lead to withdrawal from the relation or avoidance behavior rather than retaliation. Research shows that withdrawal is one of possible reactions after rejection (Smart Richman & Leary, 2009).

The hypothesis about main effect of P-IR on retaliatory aggressive behavior was not confirmed in Study 2. Instead, without ego-depletion, high P-IR was related to less aggressive behavior after rejection than low PIR (1.5 vs. 3 points more negative evaluation of a peer comparing to acceptance condition). Some explanation for this result, as in Study 1, comes from the cognitive perspective of the high P-IR person. Aggressive behavior is normative mean of improving one's mood or self-esteem for high P-IR people. Thus, other's antagonistic behavior (rejection) might not be referred to the self, but rather considered as being guided by some other forces or goals. In other words high P-IR participants could cognitively distance themselves from the rejecting behavior of a peer seeing this behavior as a consequence of a peer's bad mood, boredom or other internal factor that was not related to actual behavior of a participant. However, ego-depletion derogated the capacity for such rational evaluation of partner's motivation and disposed participants to more automatic and less rational reaction, that was retaliatory aggression.

## General Discussion

Results of both studies were not completely in line with our predictions; this suggests that the problem of mechanisms for aggressive behavior needs more research or maybe different procedures mainly for the ostracism and rejection manipulation and aggression measures should be implemented.

Exclusion procedure by Cyberball, conducted for the first time with Polish participants proved to be effective in inducing feeling of exclusion and changing mood, however the procedure did not influence displaced aggression scores. Obviously more studies are needed to verify the effectiveness of the used procedure in Poland. The reason for the lack of the main effect for aggression might be the high suspiciousness in this particular sample or some cultural variables.

Two hypothesis referring to E-IG and C-HR were partially confirmed in our studies. We found that C-HR is more significant when people are excluded and E-IR, when people are rejected. High C-HR probably leads people to see ambiguous exclusion episodes as more antagonistic, as in DeWall et al. (2009) studies, and induces more aggressive response on the basis of cognitive hostility bias (Crick & Dodge, 1994). Rejection on the other hand produced more aggression in all participants beside those with low E-IR after ego-depletion. We think that in case of such a strong main effect of rejection on aggression, ego-depletion enhanced the effect of E-IR priming the dominant reaction (Hagger, Wood, Stiff, & Chatzisarantis, 2010) that was either aggressive outburst or inhibition.

As to the third readiness for aggression pattern, P-IR, we anticipated the main, positive effect for displaced as well as direct, retaliatory aggression. Studies showed that as it is possible that high P-IR fosters displaced aggressive behavior after less severe form of exclusion, it may actually help to inhibit retaliatory aggression after rejection, when people are not depleted of self-control probably on the basis of cognitive distancing (e.g. Larsen & Christenfeld, 2011; Lott, 2002) or external attributions (e.g. Kelley & Michela, 1980) of causes of partner's rejecting behavior. When strength of self-control was depleted high P-IR participants were not able to use their cognitive resources as easily and behaved as aggressively as others.

### Limitation and Conclusion

Although research has enriched our knowledge about conditions in which particular mechanisms of aggressive behavior operate, our conclusion are limited by a series of problems. The first problem was related to the unequal gender composition of the sample in Study 2. Gender differences in aggression and readiness for aggression are well documented (Archer, 2000; Frączek et al., 2013), so it was important to control for sex differences, which we did. Though the effect of gender was not significant in either of the studies, we cannot be sure whether the results of the Study 2 would be similar if more male participants had been included. For example worse than in other studies (e.g. Frączek et al., 2015) internal consistency of RIAI scales in Study 2 could be due to the overrepresentation of woman in the sample. Another problem is also related to gender, although not gender of participants but of interaction partner in Study 2, that was female. Participants (men and women) watched an interview of a women of their age, and next were rejected by this women. It is still to be determine experimentally how the same and different gender in interacting pairs would be related to aggression after rejection. It would be also interesting to further explore how gender of the target of aggression would shape the intensity of displaced aggression in Study 1, in which gender of the opponent in CRT was not reviled.

Another issue that should limit the conclusions was the study design that we used. Both studies were constructed to explore the situational conditions that would intensify the effect of readiness for aggression on aggressive behavior. In two separate studies we examined the effect of exclusion and rejection, but at the same time we changed the object of aggression. Doing so, we were unable to separate the individual effect of the rejection vs. exclusion from the effect of the target of aggression. In future studies implemented design of the experiments should allow for determination of individual effects.

### Conclusions

Summarizing, the results of two studies indicate that different mechanisms, emotional, cognitive and related to self might take part in shaping aggressive responding after exclusion and rejection. However, there are situational factors that are important activators of these mechanisms - the level of ambiguousness of the exclusion incident, the strength of self-control and probably also the target of aggression. Future studies should focus on determining the individual influence of situational factors explored in our studies, but also on testing the effects with different aggression measurement procedures and expanding our insight into gender variability of obtained results. Presented results may also help to construct preventive and intervention programs suited to individual differences in readiness for aggression. Programs that are directed at the mechanism might be more effective because they are more comprehensive (address the important mediators or precursors of the problem) and theory driven (Nation et al., 2003).
